# Posttransplantation Lymphoproliferative Disease Treated by Retransplantation

**DOI:** 10.1155/2020/9403123

**Published:** 2020-02-25

**Authors:** Ingerid Weum Abrahamsen, Bjørn Christer Grønvold, Else Marit Inderberg, Nadia Mensali, Jonas Mattsson, Tobias Gedde-Dahl

**Affiliations:** ^1^Department of Hematology, Oslo University Hospital, Rikshospitalet, Oslo, Norway; ^2^Department of Cellular Therapy, Department of Oncology, Oslo University Hospital, The Norwegian Radium Hospital, Oslo, Norway; ^3^Institute of Clinical Medicine, University of Oslo, Oslo, Norway; ^4^Princess Margaret Cancer Center, University of Toronto, Toronto, Ontario, Canada

## Abstract

Epstein–Barr virus- (EBV-) induced posttransplantation lymphoproliferative disease (PTLD) is a life-threatening complication following allogeneic stem cell transplantation. The main risk factor is anti-thymocyte globulin (ATG). Patients who fail first-line treatment with rituximab have a poor prognosis. Though adoptive transfer of EBV-specific T cells is a potentially effective option, it is not readily available. In this case report, the patient developed PTLD following transplantation for aplastic anemia using ATG as part of the conditioning. He failed rituximab treatment and developed graft failure. We were aware that the stem cell donor had a recent EBV infection prior to transplantation, whereas the patient most likely was EBV negative before transplant. We describe our strategy to meet the patient's urgent need for EBV-specific T cells, as well as new hematopoietic stem cells. The same donor was used for a second transplant, using peripheral blood stem cells. The conditioning used was thiotepa/busulfan/fludarabin with a single dose of cyclophosphamide after transplant as graft-versus-host disease (GVHD) prophylaxis. The EBV DNA levels fell when conditioning was started, and have been undetectable since day +15 and remained so till 18 months after transplantation. The patient is doing well. This case reports successful use of cyclophosphamide after transplantation as GVHD prophylaxis, preserving virus-specific immunity.

## 1. Introduction

Epstein–Barr virus-induced posttransplantation lymphoproliferative disease (EBV-PTLD) following allogeneic stem cell transplantation is a life-threatening disease [1]. Anti-thymocyte globulin (ATG) is a well-recognized risk factor [1]. ATG is increasingly used in graft-versus-host disease (GVHD) prophylaxis as it reduces the incidence of chronic GVHD without reducing the overall survival [2]. When using transplantation to treat acquired aplastic anemia, ATG is a standard component of the conditioning regimen [3, 4].

The first-line treatment for PTLD is rituximab [4]. Patients who do not respond to this treatment have a particularly poor outcome, a 1-year overall survival of 14.6% has recently been reported [5]. Under these circumstances, adoptive EBV-specific T-cell therapy is an effective option [6]. However, this treatment is not readily available in most centers due to regulatory and quality hurdles associated with ex vivo cell manipulation for treatment purposes.

In this case report, the patient developed EBV-induced PTLD following transplantation for acquired aplastic anemia. The patient only transiently responded to rituximab treatment and developed a secondary graft failure. We describe our strategy to meet his need for EBV-specific T cells as well as a new transplantation.

## 2. Case Presentation

A 29-year-old man underwent allogeneic stem cell transplantation for very severe acquired aplastic anemia. Bone marrow stem cells were from a 10/10 (11/12) HLA-matched unrelated donor. During the pretransplant workup, antibody testing revealed that the patient was at the limit of being positive for anti-EBV IgG antibodies. It was suspected that these antibodies had been passively infused. The donor was positive for both EBV IgM and IgG antibodies but negative for EBV DNA.

A reduced intensity conditioning regimen was used consisting of fludarabine (30 mg/m^2^) and cyclophosphamide (300 mg/m^2^, both drugs from day −6 to −3), rabbit ATG (3,75 mg/m^2^ days −4 and −3), and total body irradiation (2 Gy, day −1) [7]. Initially, the course was uncomplicated, engraftment occurred at day +18, and the patient was discharged on day +23.

**Figure 1:**
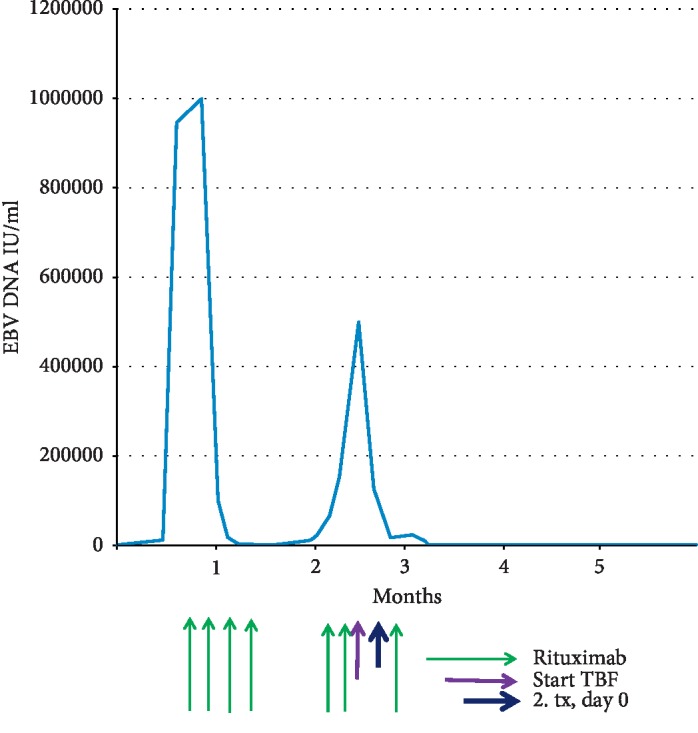
EBV-DNA/treatment. EBV DNA levels in whole blood and treatments.

The patient was readmitted on day +42 due to a parainfluenza virus infection. While hospitalized, he developed high-grade fever, generalized lymphadenopathy and splenomegaly. In parallel, whole blood EBV DNA levels increased up to 1 million IU/ml. PTLD was confirmed by biopsy, but it could not be determined whether it was mono- or polymorphic. He was treated with four doses of rituximab (375 mg/m^2^), resulting in prompt resolution of EBV levels and lymphadenopathy (Figure 1).

A few weeks later, the patient developed secondary graft failure. Unfractionated donor chimerism had been >99% on day +28 and was 16% (7% for T cells) on day +89. Concomitantly, the PTLD flared and he redeveloped high-grade fever, lymphadenopathy, and increasing EBV DNA levels, up to 500,000 IU/ml.

Based on the EBV workup before transplant, it was likely that the donor had a recent EBV infection. The same donor was selected for a second transplant as we expected the donor to have high levels of EBV-specific T cells. Directly prior to the second transplant, the donor was EBV IgM negative and IgG positive.

The conditioning regimen chosen was thiotepa (5 mg/kg days −6 and −5), busulfan (3.2 mg/kg i.v. day −4), and fludarabine (50 mg/m^2^ days −4 and −3) [8]. He was retransplanted using peripheral blood stem cells, 9.4 × 10^6^ CD34+ cells/kg recipient (day 0). GVHD prophylaxis started after transplant and consisted of one dose of cyclophosphamide (pt-Cy; 50 mg/kg, day +3), mycophenolate mofetil (1 g twice daily, days +5–28), and cyclosporine (target trough level 100–150 *µ*g/L, from day +5). The patient also received two additional doses of rituximab before the conditioning as well as one dose on day +5 (each 375 mg/m^2^) to treat the PTLD.

The patient had severe lymphadenopathy in the pharynx and neck, and on day +1, he developed acute stridor with respiratory failure and was intubated. Rectal bleeding presented on day +18, and biopsies from the colon displayed PTLD.

Engraftment started on day +14 and has been stable since. EBV DNA levels started to fall after initiation of the conditioning regimen. The EBV DNA was 500.000 IU/ml (whole blood) on day −6 (peak), 56.000 on day +1, and negative since day +15 after transplant (Figure 1). Lymphadenopathy also gradually resolved. Evaluation of donor chimerism was performed 3 months after transplant and was >99%.

**Figure 2:**
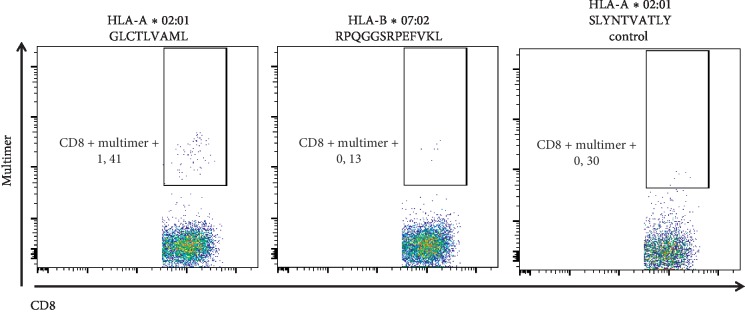
Recipient demonstrated presence of EBV-specific CD8+ T cells in peripheral blood on day +22. Peptide-MHC class I dextramer staining of peripheral blood mononuclear cells was performed for detection of CD8+ T cells specific for the immunodominant epitope from the EBV lytic protein BMLF1 (HLA-A*∗*02:01/GLCTLVAML and HLA-B*∗*07:07/RPQGGSRPEFVKL). The HLA-A*∗*02:01/SLYNTVATL dextramer (HLA-A*∗*02:01-restricted HIV gag epitope) was used as the control. T cells were gated as the CD3+, CD8+, and CD4− population cells and plots show anti-CD8 staining versus dextramer staining; HLA-A*∗*02:01/GLCTLVAML (left panel), HLA-A*∗*02:01/SLYNTVATLY (central panel), or HLA-B*∗*07:02/RPQGGSRPEFVKL (right panel).

**Figure 3:**
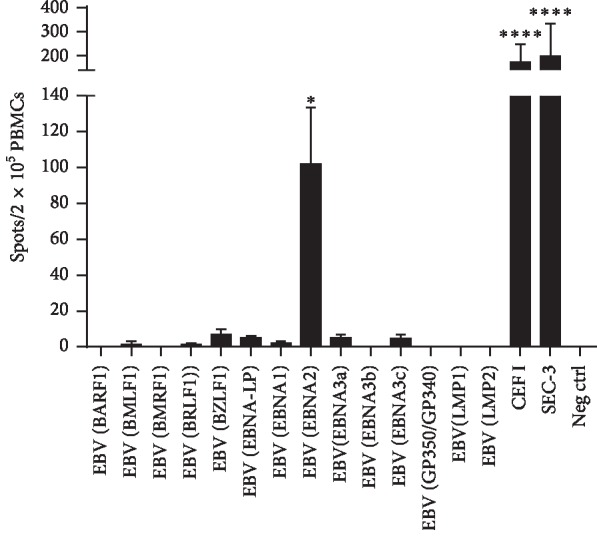
Recipient demonstrated EBV-specific IFN-*γ* secretion in PBMCs. Recipient PBMCs from day +22 were tested *ex vivo* against an overlapping 15-mer EBV peptide spanning antigenic epitopes of 14 EBV proteins in an IFN-γ ELISPOT assay. Antigenic ^*∗*^*p* < 0.05; ^*∗∗∗∗*^*p* < 0.0001.

At day +22, peripheral blood was drawn and analyzed by flow cytometry for the detection of EBV-specific T cells (Figure 2). Both donor and patient were HLA-A*∗*02:01 and HLA-B*∗*07:02 positive. A population of CD8+ T cells was found to bind an HLA-A*∗*02:01 epitope from the EBV lytic protein BMLF1, whereas no cells recognizing the HLA-B*∗*07:02 epitope from the same protein could be detected. Additionally, peripheral blood mononuclear cells were tested *ex vivo* for production of interferon-gamma (IFN-*γ*) in response to stimulation with an EBV peptide library (Figure 3). There was a very dominant response against the EBNA2 peptide pool. This was the only significant response detected, but weaker responses against several peptide pools from other proteins could also be detected.

The patient is doing well, now 18 months after transplant. Engraftment is stable, EBV DNA remains negative, and he has not developed GVHD.

## 3. Discussion

The increased risk of both PTLD (especially when using ATG) and graft rejection in transplantation of aplastic anemia is well known [9]. An additional risk factor in this patient was the EBV-serological mismatch with the donor. While this mismatch was known, the donor was considered the best available and the patient urgently needed transplantation.

Although initially effective, he failed first-line treatment of PTLD with rituximab as the PTLD rapidly relapsed. In this situation, the prognosis was dismal and therapeutic options were few. Third-party EBV-specific T cells were considered, but these were not readily available at our center. To complicate matters, the patient also had graft failure and needed a new transplantation.

While the first transplant followed a standard protocol, the second transplant was designed to optimize donor-derived EBV-specific immune responses. While bone marrow stem cells are the preferred stem cell source in acquired aplastic anemia, peripheral blood stem cells can be considered in the case of graft failure [9]. PBSC grafts contain significantly higher T-cell numbers [10] and were chosen to increase the number of EBV-specific T cells. A high stem cell dose was chosen for the same reason and in order to sustain engraftment.

The conditioning regimen was also tailored. To avoid depleting of EBV-specific T cells, ATG was avoided. Haploidentical stem cell transplantation protocols using pt-Cy report low incidences of PTLD, suggesting that EBV-specific donor immunity is preserved [11]. We therefore chose a reduced intensity haploidentical transplantation protocol using pt-Cy as our main GVHD prophylaxis.

pt-Cy has been proposed to selectively deplete proliferating, alloreactive T cells [12]. One can argue that EBV-specific T cells must have been proliferating in a similar manner as alloreactive T cells, as EBV antigens were readily available. We therefore chose to give only one dose of pt-Cy, hopefully reducing depletion of EBV-specific T cells. EBV-specific T cells were indeed detected in peripheral blood from the patient 22 days after transplantation. The safety of a single dose of pt-Cy has been shown [12]. Though recipients of a single dose of pt-Cy had a trend toward greater risk of chronic GVHD, there were no differences in treatment-related mortality. Further, our patient's donor was HLA matched and not haploidentical, which reduces the need for GVHD prophylaxis compared to the haploidentical donor setting.

Though many of the treatment decisions taken increased the risk of GVHD, this was considered acceptable in a clinical situation with few other options. A CD34+ stem cell boost combined with EBV-specific donor T cells could have been an alternative but was not considered an option in the available timeframe.

Data published by Roberto et al. suggest that antigen-specific memory T cells may survive pt-Cy and expand in the presence of the cognate antigen [13]. Further, the favorable results using pt-Cy in haploidentical transplantation has led to the investigation of pt-Cy in unrelated HLA-matched donor transplants as GVHD prophylaxis [14]. Though immune reconstitution is not yet extensively studied in this clinical setting, the clinical outcome of this case indicates its potential promise and warrants further evaluation.

## Supplementary Materials

Supplementary Materials Methods for flow cytometry and ELISPOT assays.
