# Supplementary material for: Posttransplantation Lymphoproliferative Disease Treated by Retransplantation
Source: Case Reports Immunol. 2020 Feb 25;2020:9403123. doi: 10.1155/2020/9403123 (PMC7060862; doi:10.1155/2020/9403123)
Supplement: Supplementary Materials — Methods for flow cytometry and ELISPOT assays. [file 9403123.f1.pdf]

## Supplementary material

### Flow cytometry and peptide-MHC multimer staining

Dextramers specific for EBV HLA-A*0201/GLCTLVAML, EBV HLA-B*07:02/RPQGGSRPEFVK or an irrelevant peptide HLA-A*0201/ SLYNTVATL (NEG), APC-labeled, (all from Immudex, Copenhagen, Denmark) were used for the detection of EBV-specific CD8+ T cells. PBMCs were stained according to the manufacturer's recommendation. The following antibodies were added for appropriate gating of the cells; anti-CD3-FITC (eBioscience, Thermo Fisher Scientific, Waltham, MA, USA), anti-CD4-PE-Cy7 (eBioscience), anti-CD8-BV605 (BioLegend, San Diego, CA, USA. Cells were acquired on a BD FACSCanto flow cytometer (BD Biosciences, San Jose, CA 95131, USA) and the data analyzed using FlowJo software (Treestar Inc., Ashland, OR, USA).

### Gating strategy

Cells were gated on live cells based on forward scatter (FSC) and side scatter (SSC) (gate A). CD3+ T cells (gate B) were selected and analyzed for the expression of CD4 and CD8 (gate C). CD8+ T cells were further selected and the percentage of identified multimer$^{+}$ CD8$^{+}$ T cells reported of the total number of CD8+ T cells (gate D) (see Supplementary figure 1).

### IFN-γ ELISPOT assay

IFN-γ ELISPOT assays were performed on PBMCs *ex vivo*. Anti-IFN-γ coating antibody was purchased from Mabtech (Nacka Strand, Sweden) and used to coat MultiScreenHTS HA filter plates (Merck Millipore Burlington, MA, USA). PBMCs were tested against overlapping 15-mer peptide library PepMix™ Collection EBV (JPT Peptide Technologies, Berlin, Germany) with 14 peptide pools spanning the corresponding antigenic epitopes. Negative controls with 1% DMSO and positive controls with SEC-3 superantigen (0.1µg/ml) (Toxin Technology Inc, USA) and CEF I PepMix™ Peptide Pool (JPT

Peptide Technologies) were included. PBMCs were seeded at $2x10^5$ cells per well and all conditions were tested in triplicates. Plates were incubated for 24 hours prior to the addition of detection reagents anti-IFN-γ-biotin and streptavidin-alkaline phosphatase enzyme conjugate (both from Mabtech) and BCIP/NBT substrate (Sigma-Aldrich, Saint-Louis, Missouri, USA). Spots were enumerated using an automated analyzer, CTL IMMUNOSPOT S5 VERSA-02-9030 (Cellular Technology Ltd, Shaker Heights, USA). Specific spots were calculated by subtracting the mean number of spots + (2×SD) of the medium-only control from the mean number of spots of experimental wells.

**Statistical analysis**

Statistics were made with 1-way ANOVA (multiple comparisons) with Dunnett correction using GraphPad Prism version 6.0 (GraphPad Software, San Diego, CA, USA). * $p<0.05$, ** $p<0.01$, *** $p<0.001$, **** $p<0.0001$.

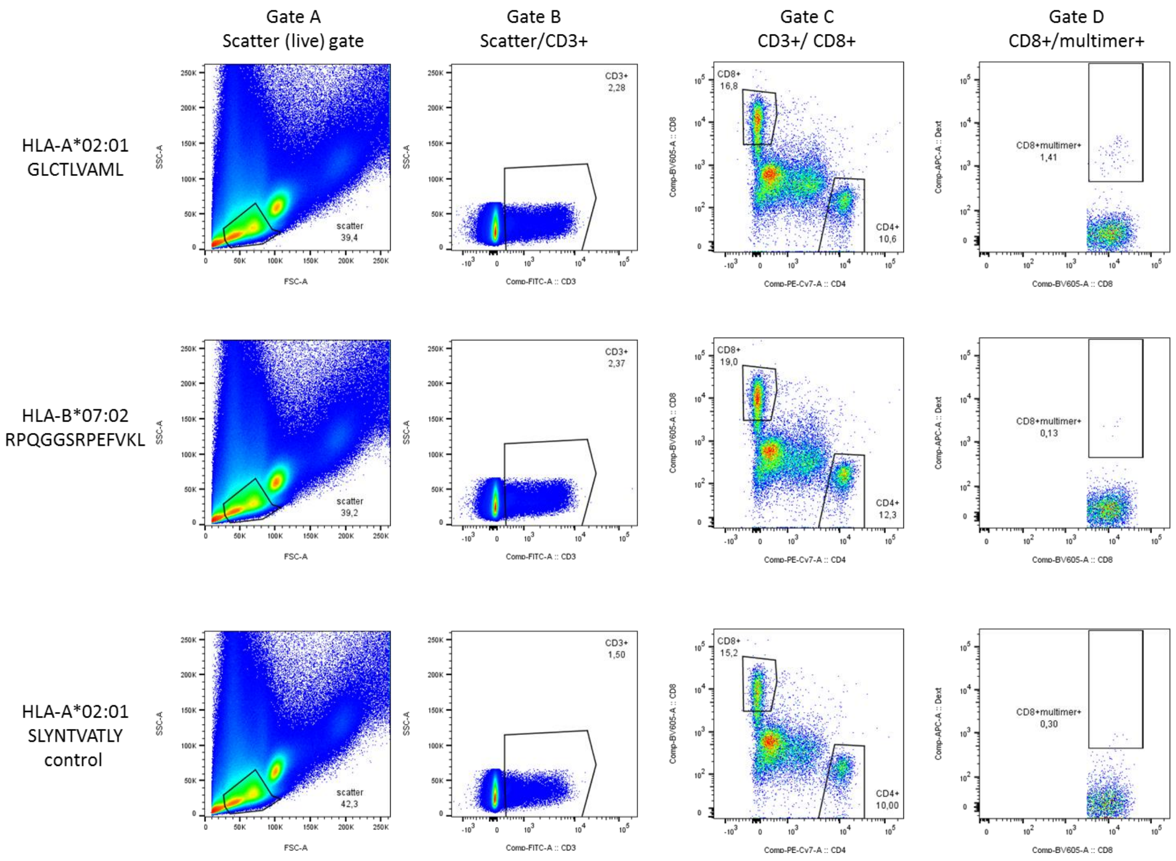


**Supplementary figure 1. Gating strategy for detection of EBV-specific CD8+ T cells.**

Peripheral blood mononuclear cells were gated on live cells based on forward scatter (FSC) and side scatter (SSC) (gate A). CD3+ T cells (gate B) were selected and analyzed for the expression of CD4 and CD8 (gate C). CD8+ T cells were further selected and the percentage of identified multimer$^+$ CD8$^+$ T cells reported of the total number of CD8+ T cells (gate D).
